# Moving from theory to practice: implementing a prehabilitation program before gastrointestinal cancer surgery (PREHAB-GI)

**DOI:** 10.1007/s00520-025-09496-5

**Published:** 2025-05-08

**Authors:** Kristy-Lee Raso, Michael Suen, Sam Egger, Jane Turner, Sonia Khatri, Yanlan Lin, Carolyn Wildbore, Caoimhe Scales, Shannon Gerber, Kin Yin Carol Chan, Guillermo Becerril-Martinez, Philip Le Page, Sim Yee Cindy Tan, Janette Vardy

**Affiliations:** 1 Department of Nutrition and Dietetics, Concord Repatriation General Hospital, Sydney, New South Wales Australia; 2 Sydney Medical School, University of Sydney, Sydney, New South Wales Australia; 3 Department of Colorectal Surgery, Concord Repatriation General Hospital, Sydney, New South Wales Australia; 4 The Daffodil Centre, The University of Sydney, A Joint Venture with Cancer Council NSW, Sydney, New South Wales Australia; 5 Concord Cancer Centre, Concord Repatriation General Hospital, Sydney, New South Wales Australia; 6 Department of Upper Gastrointestinal Surgery, Concord Repatriation General Hospital, Sydney, New South Wales Australia

**Keywords:** Prehabilitation, Implementation, Gastrointestinal cancer, Surgery, Exercise, Nutrition

## Abstract

**Purpose:**

Surgery remains the primary treatment for early-stage colorectal and upper gastrointestinal (UGI) cancers. However, it can lead to postoperative complications and reduced functionality. Prehabilitation aims to improve functional reserves before surgery. We aimed to evaluate the implementation of a multimodal prehabilitation program in “real-world” patients undergoing gastrointestinal cancer surgery.

**Methods:**

An implementation study evaluating prehabilitation in patients undergoing gastrointestinal (colorectal or UGI) cancer surgery at Concord Hospital. The prehabilitation program included supervised exercise, nutrition and nursing support delivered face-to-face or by telehealth (COVID-19 adaptations). Assessments: baseline, pre-surgery and 30 days after surgery. Primary outcome: implementation using the RE-AIM (Reach/Effectiveness/Adoption/Implementation/Maintenance) framework. Secondary outcomes: functional, nutritional and surgical outcomes, with comparisons to historical controls.

**Results:**

Between January 2020 and December 2021, 181 patients were screened; 91 (50%) were eligible. Reach: 77/91 recruited (63 colorectal, 14 UGI). Median age, 70 years (IQR, 59–79); 60% were males. Median intervention duration, 16 days (IQR, 12.25–19.75). Effectiveness: quality of life, anxiety and functional capacity improved from baseline to pre-surgery (6-min walk test (+16.1 m, *p*=0.038) and 2-min step test (+10.0 steps, *p<0.001*)). Compared to historical controls, hospital length of stay was reduced by 2.1 days (*p*=0.010), with no differences in complications. Adoption: 91% of referrals came directly from surgeons. Implementation: 94% completed the intervention, with high adherence and satisfaction levels. Maintenance: after study completion, the program was incorporated into standard care with some modifications.

**Conclusions:**

Prehabilitation can be implemented in a real-world setting, with a trend towards improving functional and surgical outcomes, but dedicated resources are necessary to implement and maintain the program.

**Supplementary Information:**

The online version contains supplementary material available at 10.1007/s00520-025-09496-5.

## Introduction

Up to 80% of colorectal [1] and 10–20% of upper gastrointestinal (UGI) cancer patients (including cancers of the stomach, liver, oesophagus, pancreas and gallbladder) [2] will undergo elective surgery, providing a chance of cure and long-term survival. Despite advances in surgery, 30–45% of gastrointestinal cancer surgery patients experience major complications [3], leading to longer hospital stays, increased costs, delayed recovery, reduced quality of life (QOL) and survival rates [4]. Even without complications, many will experience a decline in functional capacity and fail to return to their previous level of function [5].

Prehabilitation aims to improve a patient’s physical condition and overall health before surgery to enhance surgical outcomes and recovery [6]. Initially focused on exercise only [6], prehabilitation has expanded into a multimodal approach consisting of exercise, nutrition and psychosocial interventions working synergistically together to produce more favourable outcomes [7]. While controlled trials in gastrointestinal cancer patients have shown promising results [8], little is known about its integration in the “real world” with factors such as uptake, acceptance, adherence and resources often not explored.

Traditionally, randomised controlled trials are considered the highest level of evidence to guide clinical practice [9]. However, only a small proportion (14%) of published evidence are successfully incorporated into clinical care [10]. Implementation frameworks, such as RE-AIM (Reach, Effectiveness, Adoption, Implementation, Maintenance) help to identify the barriers and facilitators necessary to integrate research findings into practice [11], and can be instrumental in determining how interventions become integrated into real-world clinical care by documenting current practices, challenges and enablers. This study aims to evaluate the implementation of a multimodal (supervised exercise, nutrition and nursing support) prehabilitation program into standard care for patients undergoing curative intent gastrointestinal (colorectal or UGI) cancer surgery using the “RE-AIM” framework—evaluating Reach, Effectiveness, Adoption, Implementation and Maintenance [11].

## Materials and methods

The study design, intervention and outcomes are described in the study protocol [12]; a summary is presented below.

### Study design and setting

This is a prospective, single-centre implementation study using a before-and-after intervention design. Patients undergoing curative intent colorectal or UGI cancer surgery at Concord Repatriation General Hospital, a tertiary teaching hospital in Sydney, Australia, between January 2020 and December 2021, were invited to participate. Due to COVID-19 disruptions, recruitment was paused in March 2020 and resumed in August 2020 with telehealth adaptations. The study end date was extended from December 2020 to December 2021. The study was approved by the Sydney Local Health District Human Ethics Review Committee and conducted following the principles of the Declaration of Helsinki (2013). It was prospectively registered on the Australian and New Zealand Clinical Trials Registry (ACTR:12620000409976).

### Participants

Patients were referred by their surgeon or screened in the hospital’s preadmission clinic. Patients were eligible if they were scheduled to undergo curative intent colorectal or UGI cancer surgery at Concord Repatriation General Hospital, were ≥18 years of age, had at least 14 days before surgery, were agreeable to the exercise and nutrition interventions and were medically cleared to exercise. A professional healthcare interpreter was provided to assist non-English-speaking patients to ensure inclusivity. Patients who were unable to provide informed consent, follow instructions due to cognitive impairment or were currently receiving neoadjuvant chemotherapy or radiotherapy were ineligible. Written informed consent was obtained prior to enrolment.

### Multimodal prehabilitation intervention

Participants received a multimodal intervention for a minimum of 2 weeks before surgery, incorporating the following:

#### Exercise

The exercise program included supervised 60-min sessions, one to two times per week, at the Concord Hospital Sydney Cancer Survivorship Gym, delivered by exercise physiologists. Attending the second weekly session was optional but encouraged. Sessions were face-to-face or by telehealth (COVID-19 adaptation), individually or in small groups. Each session consisted of 20–30 min of individualised moderate-high intensity aerobic exercise (cycle ergometer, treadmill, rowing ergometer, elliptical trainer or boxing), followed by two sets of 8–12 repetitions of resistance exercise (body or hand weights, resistance bands or cable machines). Home-based aerobic and resistance exercises were prescribed for at least 30 min, 5 days/week. Exercise type, frequency and intensity were recorded in a daily diary.

#### Nutrition

Following a nutrition assessment, a dietitian provided individualised dietary education based on healthy eating principles [13], with a protein intake of 1.2–2 g/kg/body weight/day (or adjusted in overweight/obese participants) [14]. Additional dietary support was provided to manage nutrition-impact symptoms. High-protein nutritional supplements (Fresubin Protein Energy Drink, Fresenius Kabi) containing 20 g of protein were provided; one bottle was recommended daily within 60 min after exercise to support muscle synthesis [15]. Protein supplement intake was recorded in the daily diary.

#### Nursing support

Specialist cancer nurses provided weekly support, either in person or by phone, using semi-structured questions focusing on reassurance and adherence to the exercise and nutrition intervention. If necessary, referrals were made to general practitioners for medical optimisation, and smoking cessation was encouraged.

#### Miscellaneous

Distress was measured using the single-item distress thermometer [16]. Participants with a baseline score >4, indicating high psychological distress, were offered a referral to a clinical psychologist.

### Resources

Additional resources were needed for the program, including a 1-year, part-time (3 days/week) dietitian position coordinating the program and a part-time (1 day/week) exercise physiologist position. Existing hospital facilities and nursing resources were utilised.

### Postoperative care

After surgery, participants received standard postoperative care determined by their clinical team, including an enhanced recovery after surgery (ERAS) pathway for colorectal patients.

### Outcomes

The primary outcome was implementation based on the RE-AIM evaluation framework: Reach, Effectiveness, Adoption, Implementation and Maintenance [11] (Table 1).

**Table 1:** RE-AIM framework outcomes adapted to the PREHAB-GI study

Domain	Description
Reach	Number (and proportion) of eligible patients having gastrointestinal cancer surgery who participated in the study compared to eligible patients who did not consent, and patient characteristics
Effectiveness	Changes in functionality, nutrition and well-being from pre- to post-intervention. Postoperative outcomes including complications and length of hospital stay
Adoption	Number (and proportion) of surgeons who referred, and surgeon characteristics compared to non-participating surgeons. Total number of referrals, referral sources, barriers to referrals. Clinician perceptions
Implementation	Delivery of the intervention as per the protocol and adaptations made. Overall completion rates, withdrawals and reasons, adherence to exercise sessions (attendance), protein supplement consumption (recorded in daily diary) and nurse support calls completed recorded in the attendance logs or daily diary. Program evaluations
Maintenance	Status of the program 12 months after study completion and adaptions made

Secondary outcomes included changes in functional capacity (including the 6-min walk test (6MWT) and 2-min step test), nutritional status, body composition, anxiety/depression scores, QOL, and post-surgical outcomes, including hospital length of stay and complications graded using the Clavien-Dindo classification [17]. Major complications were defined as a Clavien-Dindo grade ≥3.

### Historical control group

To evaluate the effectiveness of the prehabilitation intervention, post-surgical outcomes were compared to a historical control group of similar patients who underwent elective gastrointestinal cancer surgery at the same hospital 1 year earlier (in 2019). The control group did not receive a prehabilitation intervention. Data for the control group was collected from the hospital’s colorectal cancer database or medical records (Supplementary Table 1). Functional, nutritional or psychological data were unavailable.

### Study assessments

Data was collected at three time points—baseline, pre-surgery (1–4 days before hospital admission or surgery) and 30 days (±7 days) after surgery—and managed using a project-specific Research Electronic Data Capture Database (REDCap) (Vanderbilt University) [18], hosted by Sydney Local Health District. The following variables were collected (Supplementary Table 1):

#### Participant demographic and clinical data

Demographic and clinical data (surgical procedures, hospital length of stay, 30-day readmission and complications) were collected from hospital medical records and study questionnaires.

#### Functional data

Functional capacity was measured using the 6MWT [19], 2-min step test [20], 30-s sit-to-stand [20] and handgrip strength (JAMAR hydraulic hand dynamometer).

#### Anthropometry and nutritional indicators

Height (metres) was measured using a stadiometer. Body composition (weight, fat mass, skeletal muscle mass and visceral adiposity) was assessed using bioimpedance analysis (Seca mBCA 515 Analyser (Seca, Hamburg, Germany)). Nutritional status was determined using the validated Patient-Generated Subjective Global Assessment (PG-SGA) [21] tool and categorised as well-nourished (PG-SGA A) or malnourished (PG-SGA B or C).

#### Patient-reported outcomes

QOL was assessed using the European Organisation for Research and Treatment of Cancer Quality of Life Questionnaire (EORTC QLQ-C30) [22]. Psychological well-being was evaluated using the Hospital Anxiety and Depression Scale (HADS) [23] and a single-item distress thermometer [24]. Self-reported exercise was measured using the modified Godin Shephard Leisure Time Physical Activity Questionnaire [25].

#### Satisfaction

Participants completed an investigator-developed satisfaction survey after the prehabilitation intervention (1–4 days before surgery) and 30 days after surgery. Surgical staff, including gastrointestinal surgeons, surgical ward nurses and anaesthetists, completed a post-study evaluation survey. Semi-structured qualitative interviews were conducted by an independent researcher on a subset of participants; these findings will be presented separately.

#### Adherence

Adherence to the prehabilitation program was measured by recording the number of supervised exercise sessions attended, protein supplements consumed and nursing support sessions received in the daily diary or attendance logs.

### Telehealth adaptions

The program was modified to include telehealth services in response to the COVID-19 pandemic (Table 2).

**Table 2:** Telehealth adaptations

Outcome or intervention	Telehealth amendments
Consent	Electronically completed consent forms
Baseline, pre-surgery and 30 days after surgery assessments	Assessments conducted virtually over videoconferencing/zoom appointments with links emailed to patients
Nutrition assessment	Anthropometric measures self-measured: weight, height and girths (waist, hip and mid-arm; standardised tape measure provided)
	Body composition analysis *omitted*
Exercise assessment	Remote functional capacity assessment:
	6-min walk test (6MWT) and handgrip test *omitted*
	Continuation of 30-s chair stand, 2-min step test, 3-m timed up and go (to measure cardiovascular fitness)
Supervised exercise intervention	Home-based individual supervised exercise sessions conducted virtually via videoconferencing/zoom
	Principles of the original protocol followed in terms of frequency, intensity, time and types of exercise
Nutrition intervention	Dietary education material and high-protein oral nutritional supplements mailed to patients
Patient-reported outcome/s questionnaires	Completed by hard copy (mailed out) or online/electronically using REDCap ^a^

^a^*REDCap*, Research Electronic Data Capture Database

### Statistical analysis

Descriptive statistics were used to analyse demographic and clinical data and presented as means and standard deviations, medians and interquartile ranges or counts and percentages. Generalised Estimating Equations (GEE) [26] were used to model changes from baseline for patient-reported outcome measures. A Gaussian distribution with an identity link to estimate mean differences (MD) was used for continuous outcomes, while a Poisson distribution with a log link was used to estimate relative risks (RR) for dichotomous outcomes. Robust standard errors and an independent working correlation structure were used in the GEE analyses. The GEE framework was chosen for its ability to effectively accommodate missing observations for participants under the assumption that data are missing at random. 

Differences between the prehabilitation intervention and historical control group were assessed using linear regression (reporting MD and 95% confidence intervals (CI)) for continuous outcomes and logistic regression (reporting RR and 95% CI) or chi-square (*χ*^2^) for dichotomous variables. All models were adjusted for age, gender and surgeon. Statistical analysis was performed using STATA version 18 (StataCorp. 2023. Stata Statistical Software: Release 18. College Station, TX: StataCorp LLC.). A *p*<0.05 was considered statistically significant.

## Results

**Fig. 1:**
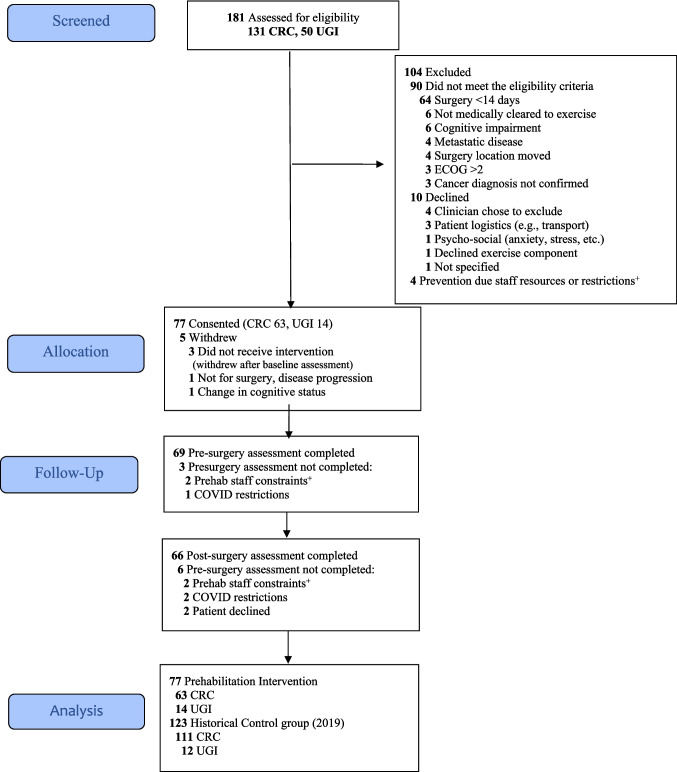
Study consort. ^**+**^Intervention or assessment unable to be completed due to staff illness/leave, COVID staff redeployment, etc. Abbreviations: CRC, colorectal cancer; UGI, upper gastrointestinal cancer

Between January 2020 and December 2021, 77 patients participated in the study (Fig. 1). Recruitment was paused in March 2020 due to COVID-19 restrictions and restarted in August 2020, with telehealth adaptations.

### Participant characteristics

Demographic and clinical data are presented in Table 3. Sixty-three (82%) had colorectal and 14 (18%) had UGI cancer. The median age was 70 years (IQR, 59–79). Age, gender and cancer type did not differ between the prehabilitation participants (*n*=77) and eligible patients who could not participate or declined (*n*=14) (Supplementary Table 2).

**Table 3:** Baseline characteristics

Characteristic	Prehab-GI (*n*=77)
Age, median (IQR), years	70 (59–79)
Sex, male, *n* (%)	46 (60)
NESB requiring an interpreter, *n* (%)	21 (27)
Smoking status, *n* (%)	
Non-smoker	47 (61)
Ex-smoker	26 (34)
Current smoker	4 (5)
Comorbidities, *n* (%)	
Diabetes	16 (21)
Hypertension	39 (51)
Cardiovascular	5 (6.5)
Pulmonary	2 (2.5)
Charlson Comorbidity Index Score ^a^, median (IQR)	5 (3.5–6)
ECOG performance status, *n* (%)	
0	70 (91)
1	7 (9)
3	0 (0)
Cancer type, *n* (%)	
Colorectal	63 (82)
Upper gastrointestinal	14 (18)
Received neoadjuvant therapy	9 (12)
Surgical approach, *n* (%) ^b,c^	
Open surgery	16 (22)
Laparoscopic	56 (78)
Type of surgery, *n* (%) ^b,c^	
Colon	42 (58.3)
Rectal	18 (25)
Gastric	6 (8.3)
Oesophageal	1 (1.4)
Pancreas	1 (1.4)
Liver	4 (5.6)
Stoma created, *n* (%) ^b,c^	12 (16.7)

*IQR* interquartile range, *NESB* non-English speaking background, *ECOG *Eastern Co-operative Oncology Group

^a ^Scores range from 0 to 24. Higher scores indicate a higher mortality risk within one year and more severe comorbid conditions

^b ^Measured at 30 days

^c ^Excludes participants who withdrew (*n*=5)

The median prehabilitation intervention (time between baseline and pre-surgery assessment) was 16 days (IQR, 12.25–19.75; range, 8–35). Four participants had an intervention period of <14 days because the surgery date was moved forward (*n*=1), work commitments (*n*=1) or interpreter unavailablity (*n*=2).

Results relating to each domain of the RE-AIM framework [11] are presented below.

#### Reach

Over 16 months, 181 (131 colorectal, 50 UGI) patients were screened (Fig. 1). Of these, 90 (50%) patients were ineligible; reasons included inadequate time before surgery (<14 days) (*n*=64, 71%) and not medically cleared to exercise (*n*=6, 7%). Among the 91 (50%) eligible patients, 10 (11%) declined to participate. Four eligible patients could not participate due to staffing issues (e.g., illness/leave, COVID-19 redeployment). Age, gender and language did not affect participation; however, colorectal cancer patients were more likely to participate.

#### Effectiveness

**Fig. 2:**
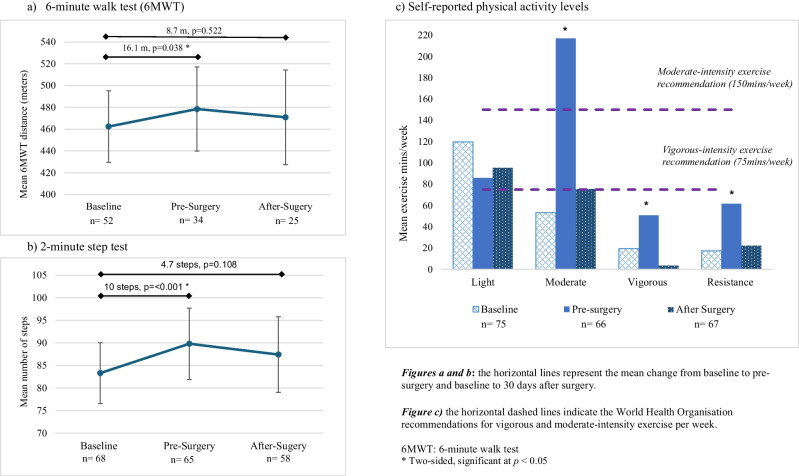
Functional capacity and exercise behaviours

Functional outcomes are presented in Table 4, Fig. 2 and Supplementary Table 3. Before-to-after intervention assessments showed positive changes in functional capacity using the 6MWT (MD, +16.1 m, *p*=0.038) and 2-min step test (MD, +10 steps, *p* ≤ 0.001). Some improvement was maintained at 30 days after surgery, although it was no longer statistically significant. However, the 6MWT could not be completed on 110 (50%) occasions. Reasons included COVID-19 restrictions and the move to telehealth (*n*=86), adverse weather conditions (outdoor track) (*n*=13), pre-existing medical conditions contraindicating participation (*n*=6), inappropriate footwear (*n*=3) or participant declined (*n*=2). There were no significant changes in other functional measures.

**Table 4:** Functional, nutritional and psychological well-being outcome measures

	Baseline, *n* =77	Pre-surgery, *n* =72	30 days after surgery, *n* =72	Change from baseline to pre-surgery		Change from baseline to after surgery	
				Mean change ^a^ (95% CI)	*p*-value	Mean change ^a^ (95% CI)	*p*-value
Functional assessment							
6-minute walk test (6MWT) (metres), mean (SD)	462.4^d^ (118.30)	471.7^d^ (110.70)	482.0^d^ (105.34)	16.1 (0.9, 31.4)	0.038*	8.7 (−17.8, 35.2)	0.522
Missing	25	38	47				
2-minute step test (steps), mean (SD)	83.3 (26.43)	89.8 (30.77)	87.4 (32.61)	10.0 (6.0, 14.0)	<0.001*	4.7 (−1.0, 10.4)	0.108
Missing	9	7	14				
Handgrip strength—right (kg), mean (SD)	32.3 (12.04)	32.7^d^ (12.49)	32.9^d^ (12.72)	1.0 (0.0, 1.9)	0.058	−0.8 (−1.8, 0.6)	0.092
Missing	17	29	41				
Handgrip strength—left (kg), mean (SD)	30.5 (11.03)	30.7^d^ (16.35)	31.2^d^ (11.03)	0.4 (−0.5, 1.3)	0.409	−1.3 (−2.3, −0.4)	0.004*
Missing	17	29	41				
Nutritional assessment							
Weight (kg), mean (SD)	71.7 (18.42)	72.2 (18.46)	68.9 (17.65)	0.5 (0.2, 0.8)	0.002*	−2.4 (−3.1, −1.8)	<0.001*
Missing	0	3	6				
Body Mass Index (BMI) (kg/m^2^), mean (SD)	24.8 (5.45)	25.5 (5.16)	24.5 (4.66)	0.4 (−0.6, 1.4)	0.448	−0.5 (−1.4, 0.4)	0.265
Missing	0	3	6				
Nutrition status							
Malnourished (PG-SGA B or C), *n* (%)	15 (19.5)	7 (10)	25 (38.2)	0.57^e^ (0.36, 0.89)	0.015*	1.98^e^ (1.23, 3.17)	0.005*
PG-SGA score, mean (SD)	6.4 (3.75)	4.2 (1.46)	6.2 (4.26)	−1.7 (−2.2, −1.1)	<0.001*	1.8 (0.6, 2.9)	0.003*
Missing	0	3	6				
Psychological well-being							
Distress thermometer	7.3 (2.9)	8.1 (2.5)	8.4 (2.5)	0.7 (0.1, 1.2)	0.016	1.1 (0.3, 1.8)	0.006
Missing	2	5	5				
HADS—anxiety, mean (SD)^b^	5.8 (3.76)	4.7 (4.23)	4.5 (3.49)	−1.1 (−1.8, −0.3)	0.004*	−1.3 (−2.2, −0.4)	0.004*
Anxiety >4, *n* (%)	47 (62.6)	32 (47.8)	28 (41.8)	1.3 (1.0, 1.8)	0.053^e^	1.5 (1.1, 2.1)	0.010^e^*
HADS- depression, mean (SD)^b^	4.1 (4.0)	3.5 (3.61)	4.4 (4.39)	−0.5 (−1.2, 0.2)	0.174	0.4 (−0.8, 1.5)	0.528
Depression >4, *n* (%)	26 (34.7)	24 (35.8)	25 (37.3)	1.0 (0.6, 1.5)	0.885^e^	0.9 (0.6, 1.4)	0.742^e^
Missing	2	5	5				
Quality of life: EORTC QLQ-C30, global health, mean (SD)^c^	65.2 (21.54)	71.8 (19.19)	64.2 (22.14)	6.2 (1.8,10.7)	0.006*	−1.7 (−0.8, 4.5)	0.583
Missing	2	5	5				

*SD* standard deviation, *6MWT* 6-minute walk test, *kg* kilograms, *PG-SGA* Patient-Generated Subjective Global Assessment, *HADS* Hospital Anxiety and Depression Scale, *EORTC QLQ-C30* European Organization for the Research and Treatment of Cancer Quality of Life Questionnaire

^a^Derived from the imputation of missing data and adjustment for confounders, including age, gender, and surgeon. A Gaussian distribution estimated mean differences for continuous outcomes, and a Poisson distribution estimated relative risks for dichotomous outcomes

^b^Scores range from 0–21, with higher scores indicating greater levels of anxiety or depression. A score >8 indicates high symptoms of anxiety or depression

^c^EORTC QLQ-C30—Global health: A higher score indicates a better quality of life and functioning

^d^Indicates >30% of participants missing

^e^Relative risks

*Two-sided, significant at *p *< 0.05

At baseline, 15 participants (19.5%) were malnourished (PG-SGA B/C) (Table 4 and Supplementary Table 3). UGI participants had higher incidence of malnutrition compared to colorectal participants (colorectal, 17.5% vs. UGI, 28.6%, *p*=0.455) (Supplementary Table 3). There were no significant differences in characteristics and surgical outcomes between well-nourished and malnourished participants. PG-SGA scores, an indication of nutritional-related risk, improved from baseline to pre-surgery with prehabilitation (baseline, 6.4 vs. pre-surgery, 4.2, MD −1.7 (−2.2, −1.1), *p* ≤ 0.001). No significant changes were seen in body composition measures (Supplementary Table 3).

Patient-reported outcomes are outlined in Table 4 and Supplementary Table 3. Global health-related QOL, emotional, fatigue, dyspnoea subscales and anxiety symptoms significantly improved from baseline to pre-surgery. Of these, only the improvements in anxiety symptoms were maintained 30 days after surgery.

Length of hospital stay was 2.1 days shorter for prehabilitation participants compared to historical controls (prehabilitation, 6.9 days; SD, 5.8, vs. historical control, 8.8 days; SD, 7.3, *p*=0.010) (Table 5). Colorectal participants had a shorter length of stay than UGI participants (5.8 vs. 11.9 days, *p*=0.062). Thirty (42%) participants experienced at least one complication (Table 5). No differences were seen in the number and severity of complications, hospital readmission rates, unplanned intensive care unit admissions or discharge destination.

**Table 5:** Postoperative outcomes—Prehab-GI intervention vs. historical control groups

Operative outcomes	Prehab-GI, *n *=72	Historical control group (2019), *n *= 123	Adjusted estimates ^a^ (95% CI)	*p -*value
Complications, *n* (%) ^b^				
Total (at least 1 complication)	30 (41.7)	52 (42.3)	RR, 1.0 (0.7, 1.4)	0.933
Severe, Clavien-Dindo grade ≥3	6 (8.3)	14 (11.4)	RR, 0.6 (0.2, 1.4)	0.226
Length of hospital stay (days), mean (SD) ^b^	6.9 (5.6)	8.8 (7.3)	MD, −2.1 (−3.6, −0.5)	0.010*
Unplanned ICU admissions, *n* (%)	4 (5.6)	8 (6.6)	RR, 0.8 (0.7, 2.7)	0.791
Hospital readmission, *n* (%) ^b^	6 (8.3)	9 (7.3)	RR, 1.1 (0.4, 3.1)	0.707
Discharge destination, *n* (%) ^b^				
Home	71 (98.6)	98 (79.7)		0.0002*
Rehab	1 (1.4)	25 (20.3)		

*SD* standard deviation, *ICU* intensive care unit, *MD* mean difference, *RR* relative risk

^a^Adjustment for confounders, including age, gender and surgeon. Continuous outcomes were assessed using linear regression (MD) and dichotomous variables using logistic regression (RR or χ^2^)

^b^Measured at 30 days

*Two-sided, significant at *p *< 0.05

#### Adoption

Fourteen surgeons were invited to participate: eight colorectal and six UGI. All invited colorectal surgeons participated. Among the UGI surgeons, two did not operate at the hospital during the study period, and one did not refer to the study. Of the referrals, 70 (91%) came directly from surgeons, while 7 (9%) were identified through the preadmission clinic. Referrals fluctuated throughout the study period due to  COVID-19 disruptions including cancellation of elective surgeries. (Supplementary Figure 1). Following completion of the study, 10 of the 11 (91%) participating surgeons incorporated prehabilitation into their standard care. An additional surgeon who was not involved in the study adopted the program. All surveyed clinicians reported the program helped patients prepare for surgery and would highly recommend it.

Clinician 7: *“A great initiative, a no-brainer. The benefits go way beyond shortening hospital stay”*.

Clinician 6: *“What a great program. Patients love it and do so well”*.

#### Implementation

All participants (*n*=77) completed their baseline assessment. Five withdrew because of psychosocial stressors (*n*=3), change in cognition *(n*=1) and cancellation of surgery due to disease progression (*n*=1). Completion rates for pre-surgery (96%, 69/72) and 30 days after surgery (92%, 66/72) assessments were high. However, some participants could not complete their assessments due to prehabilitation staff unavailability *(n*=4), COVID-related restrictions (*n*=3) or participant decline (*n*=2). Overall, 35% (74/212) of assessments were conducted via telehealth.

Most participants attended one supervised exercise session per week, with two participants attending the second optional exercise session. Approximately one-third (32%) of the supervised exercise sessions were delivered through videoconferencing. No serious adverse events were reported. 

Adherence to the study was high: 85% (61/72) of participants attended the available supervised exercise sessions, 90% (65/72) consumed the prescribed high-protein supplements, and 93% (67/72) received the provided nurse calls. Reasons for nonadherence included time constraints with medical appointments (*n*=5 exercise), exercise contraindications (*n*=1), forgetfulness (*n*=5; 3 nutrition, 2 exercise), participant declined (*n*=5; 3 exercise, 2 nursing), supplement tolerance (*n*=4) and interpreter unavailability (*n*=3 nursing). On 22 occasions, the supervised exercise sessions or nursing calls could not be delivered due to staff unavailability (e.g., illness/leave, COVID-19 disruptions). Cancer type, age, gender, language, employment status and surgeon did not affect adherence. Nearly all (96%) surveyed participants were strongly satisfied or satisfied with the program and would recommend it to others.

P12: *“Very organised, it kept me motivated before surgery. I really enjoyed the Zoom sessions”*.

P34: *“I feel stronger and happier”*

P70: *“The support was great! It got me ready for my surgery.”*

P22: *“A fantastic program; the staff were exceptional.”*

#### Maintenance

After completing the study, some modifications were made to the prehabilitation program to ensure its sustainability. Complementary nutritional supplements were no longer provided, and patients had to purchase their own. The exercise type, intensity and frequency were modified, with a change in delivery from exercise physiologists to physiotherapists due to staff availability.

In 2022, 49/117 (42%) eligible patients participated in the prehabilitation program: 34 (69%) colorectal and 15 (31%) UGI cancer. Of these, 39/49 (80%) received the exercise intervention, 44/49 (90%) received nursing support, and all 49 (100%) received the nutrition intervention.

## Discussion

National and international guidelines recommend prehabilitation as an essential part of cancer and surgical pathways [14, 27]. Despite its benefits, prehabilitation is not routinely practised. This pragmatic study demonstrates that prehabilitation can be effectively integrated into routine clinical care for patients undergoing gastrointestinal cancer surgery, using telehealth services to combat pandemic-related challenges.

Using the RE-AIM framework [11], we found that our multimodal prehabilitation program was highly acceptable and had good fidelity. Almost all eligible patients referred participated, consistent with similar prehabilitation programs [28]. However, despite our efforts to promote the program across a multidisciplinary setting, the most common reason for ineligibility was insufficient time before surgery (<14 days) (*n*=64). Hospital administrative processes and the cancellation of elective surgeries in the public health system due to COVID-19 disruptions further impacted patient recruitment. According to current cancer treatment guidelines, patients should receive primary definitive treatment, including surgery, within 31 days, with delays potentially impacting survival outcomes [29, 30]. Given this narrow window, integrating a digital dashboard with automated risk stratification in the preoperative workflow could help identify suitable patients earlier, improve recruitment and maximise the prehabilitation duration while reducing the reliance on individual clinicians for screening [31].

Despite the challenges brought about by COVID-19, we achieved high completion and adherence rates, exceeding the average reported in the literature [28]. This success can be attributed to the program’s design, individualised intervention, coordination, supervision and involvement of experienced gastrointestinal surgical and oncology multidisciplinary staff, including a dietitian, exercise physiologists and nurses. The use of telehealth in response to the COVID-19 pandemic also played a significant role ensuring continuity of care. Many participants felt a sense of control over their health and viewed the program as a positive distraction while waiting for surgery. Research suggests that peer support and supervised exercise are associated with higher adherence rates [32]. Although our program mainly relied on group-based supervised exercise sessions, it is essential for future programs to consider local resources and patient needs when designing a prehabilitation program.

In line with the literature [8, 33], we observed improvements in functional capacity, exercise behaviours and nutritional status. While improvements diminished 30 days after surgery, functional outcomes and exercise behaviours remained higher than baseline. However, the results must be interpreted cautiously as some outcomes were omitted due to COVID-19 disruptions and the transition to telehealth. Surgical nutrition and oncology exercise guidelines recommend completing a pre-intervention assessment and monitoring to ensure safety and provide guidance [14, 34]. Although virtual assessments were quickly implemented, at the time of the study, there was limited evidence supporting the use of validated-telehealth functional outcome measures, including the 6MWT. Since then, several outcome measures have emerged and recommended in a telehealth setting [35].

Compared to historical data, prehabilitation patients had shorter hospital stays. However, the impact of other confounding factors, such as COVID-19 [36], surgical complexity and a patient’s self-motivation to participate, cannot be excluded. As the pandemic evolved, many services were redirected, reducing non-urgent diagnostic and surgical procedures to prioritise emergency and high-priority elective cases. Private hospital facilities were used, enabling public patients to receive treatment earlier. Additionally, extra efforts were made to discharge patients as soon as they were medically cleared to reduce the risk of hospital-acquired COVID-19 exposure [37], likely impacting the length of stay.

Having accessible and experienced prehabilitation team members with strong relationships with surgeons or anaesthetists facilitated the adoption of prehabilitation. Almost all invited surgeons adopted the program, making up 91% of the referrals. However, the long-term success of prehabilitation relies on continued engagement and dedicated resources. Unfortunately, there was no ongoing dedicated funding for our prehabilitation service, resulting in several modifications after the study’s maintenance phase. While a formal cost-effectiveness evaluation is underway, early economic evaluations suggest that prehabilitation can save up to £3000 (AUD 5200) per person [38] due to reduced complications, shorter hospital stays, decreased intensive care needs and fewer services needed upon discharge. These potential cost savings could be used to establish, maintain and expand prehabilitation programs.

 As the demand for supporting vulnerable surgical patients increases, telehealth services are becoming a viable option, especially for the elderly or people living in rural or remote areas. While they cannot fully replace essential in-person visits, they can help reduce health inequities and improve access to services, regardless of geographical location [39]. A systematic review found that the travel distance to the exercise facility often influenced participation and adherence [40]. Interestingly, approximately 30% of our program was conducted via videoconference, with good patient acceptance. With the increasing demand for home-based or virtual prehabilitation programs, further research is needed to evaluate the safety, feasibility and effectiveness of web-based platforms, apps and wearables.

We acknowledge that our study has several limitations. Due to COVID-19 pandemic disruptions, a large amount of data for several key outcomes, including the 6MWT, is missing. To try to address this, we used the GEE method. Although we did not have a prospective control arm, we attempted to mitigate this limitation by using a historical control arm. Nevertheless, one strength of the study is that the program was implemented in a real-world clinical setting and included patients from diverse backgrounds. In response to the COVID-19 pandemic challenges, we quickly adapted the program to include a telehealth component. This adaption ensured the ongoing delivery during a time of great uncertainty, and helped overcome additional logistical barriers surgical patients may face, particularly those with work or family commitments or living far from the hospital. While the Maintenance phase of the RE-AIM framework was not extensively explored, key barriers included resources and fragmented screening/referral pathways during the transition to standard care. Further exploration of the barriers and facilitators to maintaining the program is needed from the perspectives of patients, clinicians, and institution.

## Conclusion

The healthcare system is complex, and new concepts must be tailored to local settings. We showed that a multimodal prehabilitation program can be successfully implemented in face-to-face and telehealth settings, with a trend towards improving functional and surgical outcomes. While the current program has not expanded, it has provided valuable insights for the development and implementation of other prehabilitation programs.

## Supplementary Information

Below is the link to the electronic supplementary material.

Supplementary file1 (DOCX 107 KB)

## Data Availability

Available from the corresponding author upon reasonable request.
