# Supplementary material for: Moving from theory to practice: implementing a prehabilitation program before gastrointestinal cancer surgery (PREHAB-GI)
Source: Support Care Cancer. 2025 May 8;33(6):458. doi: 10.1007/s00520-025-09496-5 (PMC12062105; doi:10.1007/s00520-025-09496-5)
Supplement: Supplementary file 1 — Supplementary file1 (DOCX 107 KB) [file 520_2025_9496_MOESM1_ESM.docx]

**Supplementary Figure 1:** Prehabilitation program referrals.

COVID-19 disruptions: Prehab-GI program on hold due to cancellation of elective surgeries

COVID-19 disruptions: Prehab-GI program on hold due to cancellation of elective surgeries

**Supplementary Table 1:** Data variables and collection time points.

| **Data Variable** | **Baseline (T0)** | **Pre-Surgery (T1)** | **Hospital admission** | **30-days after surgery (T2)** | **Historical control (2019)** |
| --- | --- | --- | --- | --- | --- |
| **Demographics**   - Age - Sex - Smoking history - Language - Medical history - Concomitant medications - Cancer diagnosis - Comorbidity index [1] - Eastern Co-operative Oncology Group performance status [2] | X  X  X  X  X  X  X  X  X | X  X |  | X | X  X  X |
| **Functional assessment**   - Handgrip strength (omitted with telehealth) - 30-second chair stand [3] - 2-minute step test [3] - 3-m timed up and go [4] - 6-minute walk test (omitted with telehealth) [5] - Activities of daily living [6] | X  X  X  X  X  X | X  X  X  X  X  X |  | X  X  X  X  X  X |  |
| **Anthropometrics and nutrition assessment**   - Height (cm) - Weight (kg) - Body mass index (BMI) (kg/m^2^) - Body composition: Fat mass, skeletal muscle mass, visceral adiposity. (omitted with telehealth) - Waist circumference (cm) - Nutritional status using PG-SGA [7] - Dietary intake (calories and protein): 24-hour diet recall | X  X  X  X  X  X  X | X  X  X  X  X  X |  | X  X  X  X  X  X |  |
| **Patient-reported outcomes**   - Exercise behaviors-Modified Godin’s Leisure Time Physical Activity Questionnaire [8] and exercise log/daily diary - QoL: EORTC QLQ-C30 [2] - Fatigue: FACIT-F subscale [9] - Symptoms of anxiety and depression- HADS [10] - Sarcopenia screening – SARC-F [11] - Distress- National Comprehensive Cancer Network Distress Thermometer (single item) [12] | X  X  X  X  X  X | X  X  X  X  X  X |  | X  X  X  X  X  X |  |
| **Surgical outcomes**   - Complications: intra and postoperative using Clavien Dindo Grading (1-V) [13] - Length of hospital stay (days) - Discharge information including discharge date, destination, social services required - Length of physiotherapy intervention (days) - 30-day readmission |  |  | X  X  X  X | X  X | X  X  X  X |
| **Compliance/adherence**   - Exercise session attendance - Home-based daily diary (self-reported) - Nursing support calls completion - Prescribed high protein oral nutritional supplement consumption (self-reported) |  | X  X  X  X |  |  |  |
| **Program evaluation**   - Participant investigator-developed program satisfaction. - Qualitative exit interview |  | X | X | X  X |  |

**Abbreviations:**
PG-SGA: Patient-Generated Subjective Global Assessment.
EORTC QLQ-C30: European Organization for the Research and Treatment of Cancer Quality of Life Questionnaire.
FACIT*-*F: Functional Assessment of Chronic Illness Therapy—Fatigue.
HADS: Hospital Anxiety and Depression Scale.
SARC-F: Strength, assistance with walking, rising from a chair, climbing stairs, and falls

**Supplementary Table 2:** Characteristics- Prehab-GI vs non-participants vs historical control.

| Characteristic | Prehab-GI (n=77) | Non-participants (n=14) | Historical control (n=123) |
| --- | --- | --- | --- |
| Age, median (IQR), years | 70 (59-79) | 69 (60.5-75) | 72 (64-81) |
| Sex, male, n (%) | 46 (60) | 9 (64) | 68 (55) |
| Cancer Type n (%)  Colorectal  Upper gastrointestinal | 63 (82) 14 (18) | 8 (57) 6 (43) | 111 (90) 12 (10) |

**Supplementary Table 3**: Functional, nutritional, and patient-reported outcome measures.

|  |  |  |  | **Change from baseline  to pre-surgery** | | **Change from baseline  to post-surgery** | |
| --- | --- | --- | --- | --- | --- | --- | --- |
|  | **Baseline n=77** | **Pre-surgery n=72** | **After surgery n=72** | **mean change ^a^ (95% CI)** | **p-value** | **mean change ^a^ (95% CI)** | **p-value** |
| **Functional assessment** |  |  |  |  |  |  |  |
| 6-minute walk test (6MWT) (meters), mean |  |  |  |  |  |  |  |
| All participants | 462.4 ^d^ | 471.7 ^d^ | 482.0 ^d^ | 16.1 (0.9, 31.4) | 0.038* | 8.7 (-17.8, 35.2) | 0.522 |
| CRC | 466.0 ^d^ | 473.2 ^d^ | 465.7 ^d^ | 13.9 (-3.4, 31.3) | 0.116 | -0.1 (-29.5, 29.3) | 0.995 |
| UGI | 447.2 | 463.0 ^d^ | 567.8 ^d^ | 28.6 (-0.6, 57.9) | 0.055 | 51.5 (9.4, 93.7) | 0.017* |
| Missing | 25 | 38 | 47 |  |  |  |  |
| CRC/UGI | 21/4 | 31/7 | 34/13 |  |  |  |  |
| 2-minute step test (steps), mean |  |  |  |  |  |  |  |
| All participants | 83.3 | 89.8 | 87.4 | 10.0 (6.0, 14.0) | <0.001* | 4.7 (-1.0, 10.4) | 0.108 |
| CRC | 82.9 | 88.5 | 86.2 | 9.9 (5.2, 14.6) | <0.001* | 4.5 (-2.0, 11.0) | 0.172 |
| UGI | 84.6 | 96.3 | 93.0 | 9.6 (3.3, 15.9) | 0.003* | 4.9 (-7.1, 16.8) | 0.425 |
| Missing | 9 | 7 | 14 |  |  |  |  |
| CRC/UGI | 8/1 | 6/1 | 12/2 |  |  |  |  |
| Hand Grip Strength Right (kg), mean |  |  |  |  |  |  |  |
| All participants | 32.3 | 32.7 ^d^ | 32.9 ^d^ | 1.0 (0.0, 1.9) | 0.054 | -0.8 (-1.8, 0.1) | 0.092 |
| CRC | 32.9 | 32.1^d^ | 32.3 ^d^ | 0.9 (-0.1, 1.8) | 0.088 | -0.7 (-1.7, 0.3) | 0.173 |
| UGI | 29.8 | 36.4 ^d^ | 37.3 ^d^ | 1.6 (-2.0, 5.1) | 0.394 | -1.7 (-4.9, 1.5) | 0.289 |
| Missing | 17 | 29 | 41 |  |  |  |  |
| CRC/UGI | 15/2 | 23/6 | 33/8 |  |  |  |  |
| Hand Grip Test Left (kg), mean |  |  |  |  |  |  |  |
| All participants | 30.5 | 30.7 | 31.2 | 0.4 (-0.5, 1.3) | 0.409 | -1.3 (-2.3, -0.4) | 0.004* |
| CRC | 30.8 | 30.4 | 30.6 | 0.6 (-0.2, 1.5) | 0.154 | -1.4 (-2.4, -0.4) | 0.008* |
| UGI | 29.3 | 32.6 | 35.6 | -1.2 (-4.5, 2.2) | 0.497 | -1.2 (-3.7, 1.2) | 0.321 |
| Missing | 17 | 29 | 41 |  |  |  |  |
| CRC/UGI | 15/2 | 23/6 | 33/8 |  |  |  |  |
| 30 second chair stand (reps), mean |  |  |  |  |  |  |  |
| All participants | 12.5 | 14.2 | 17.0 | 1.7 (0.8, 2.7) | <0.001* | 4.4 (-0.7, 9.6) | 0.093 |
| CRC | 12.5 | 14.4 | 17.9 | 1.9 (0.9, 2.9) | <0.001* | 5.3 (-0.9, 11.4) | 0.093 |
| UGI | 12.4 | 13.3 | 12.7 | 0.9 (-1.1, 3.0) | 0.383 | -0.5 (-2.2, 1.2) | 0.584 |
| Missing | 2 | 3 | 10 |  |  |  |  |
| CRC/UGI | 1/1 | 3/0 | 8/2 |  |  |  |  |
| Timed Up and Go (seconds), mean |  |  |  |  |  |  |  |
| All participants | 7.2 | 7.0 | 7.2 | -0.2 (-0.5, 0.2) | 0.311 | 0.3 (-0.2, 0.8) | 0.267 |
| CRC | 7.2 | 6.8 | 7.0 | -0.3 (-0.7, 0.1) | 0.109 | 0.0 (-0.3, 0.4) | 0.786 |
| UGI | 7.4 | 7.8 | 8.3 | 0.5 (-0.4, 1.4) | 0.277 | 1.5 (-0.9, 3.9) | 0.227 |
| Missing | 0 | 9 | 11 |  |  |  |  |
| CRC/UGI | 0/0 | 7/2 | 9/2 |  |  |  |  |
| **Nutritional assessment** |  |  |  |  |  |  |  |
| Weight (kg), mean |  |  |  |  |  |  |  |
| All participants | 71.7 | 72.2 | 68.9 | 0.5 (0.2, 0.8) | 0.002* | -2.4 (-3.1, -1.8) | <0.001* |
| CRC | 72.0 | 72.4 | 69.4 | 0.4 (0.1, 0.8) | 0.019* | -2.2 (-2.9, -1.4) | <0.001* |
| UGI | 70.2 | 71.2 | 66.7 | 0.8 (0.3, 1.3) | <0.001* | -3.7 (-5.0, -2.3) | <0.001* |
| Missing | 0 | 3 | 6 |  |  |  |  |
| CRC/UGI | 0/0 | 3/0 | 5/1 |  |  |  |  |
| BMI (kg/m2), mean |  |  |  |  |  |  |  |
| All participants | 24.8 | 25.5 | 24.6 | 0.4 (-0.6, 1.4) | 0.448 | -0.5 (-1.4, 0.4) | 0.265 |
| CRC | 24.7 | 25.4 | 24.7 | 0.3 (-0.9, 1.6) | 0.585 | -0.4 (-1.4, 0.7) | 0.505 |
| UGI | 25.1 | 25.9 | 24.1 | 0.6 (0.3, 0.9) | <0.001* | -1.3 (-1.8, -0.7) | <0.001* |
| Missing | 0 | 3 | 6 |  |  |  |  |
| CRC/UGI | 0/0 | 3/0 | 6/1 |  |  |  |  |
| Waist circumference (cm), mean |  |  |  |  |  |  |  |
| All participants | 95.2 | 95.1 | 93.8 | -0.3 (-1.0, 0.4) | 0.384 | -1.5 (-2.3, -0.7) | <0.001* |
| CRC | 95.3 | 94.7 | 93.1 | -0.5 (-1.3, 0.2) | 0.171 | -1.5 (-2.4, -0.5) | 0.002* |
| UGI | 94.9 | 97.4 | 97.1 | 1.2 (-0.4, 2.8) | 0.140 | -1.2 (-3.0, 0.6) | 0.202* |
| Missing | 1 | 6 | 13 |  |  |  |  |
| CRC/UGI | 1/0 | 4/2 | 11/2 |  |  |  |  |
| Fat mass (kg), mean |  |  |  |  |  |  |  |
| All participants | 24.6 | 24.3 ^d^ | 23.3 ^d^ | 0.2 (-0.2, 0.6) | 0.352 | -1.3 (-1.9, -0.6) | <0.001* |
| CRC | 25.3 | 24.7 ^d^ | 24.1 ^d^ | 0.2 (-0.3, 0.7) | 0.375 | -0.9 (-1.5, -0.3) | 0.003* |
| UGI | 22.2 | 21.9 ^d^ | 19.3 ^d^ | 0.1 (-1.0, 1.3) | 0.821 | -3.2 (-4.9, -1.5) | <0.001* |
| Missing | 19 | 30 | 41 |  |  |  |  |
| CRC/UGI | 17/2 | 24/6 | 34/7 |  |  |  |  |
| Skeletal muscle mass (kg), mean |  |  |  |  |  |  |  |
| All participants | 21.5 | 21.4 ^d^ | 21.0 ^d^ | 0.4 (-0.4, 1.1) | 0.330 | -1.0 (-1.4, -0.6) | <0.001* |
| CRC | 21.6 | 21.1 ^d^ | 20.9 ^d^ | 0.4 (-0.4, 1.3) | 0.342 | -0.8 (-1.2, -0.5) | <0.001* |
| UGI | 21.1 | 23.3 ^d^ | 21.5 ^d^ | 0.1 (-0.5, 0.6) | 0.835 | -1.9 (-3.1, -0.7) | 0.002* |
| Missing | 19 | 30 | 41 |  |  |  |  |
| CRC/UGI | 17/2 | 24/6 | 34/7 |  |  |  |  |
| Visceral Adiposity Tissue (Litres), mean |  |  |  |  |  |  |  |
| All participants | 3.3 | 3.1 ^d^ | 2.9 ^d^ | -0.1 (-0.2, 0.0) | 0.065 | -0.3 (-0.4, -0.1) | <0.001* |
| CRC | 3.3 | 3.0 ^d^ | 2.9 ^d^ | -0.1 (-0.2, 0.0) | 0.027* | -0.2 (-0.4, -0.1) | 0.002* |
| UGI | 3.5 | 3.6 ^d^ | 3.2 ^d^ | 0.1 (-0.1, 0.2) | 0.362 | -0.4 (-0.6, -0.3) | <0.001* |
| Missing | 19 | 30 | 41 |  |  |  |  |
| CRC/UGI | 17/2 | 24/6 | 34/7 |  |  |  |  |
| Malnourished (PG-SGA B or C) (%) |  |  |  |  |  |  |  |
| All participants | 19.5 | 10.1 | 38.2 | 0.57 (0.36, 0.89) ^e^ | 0.015* | 1.98 (1.23, 3.17) ^e^ | 0.005* |
| CRC | 17.5 | 12.3 | 31.6 | 0.75 (0.53, 1.07) ^e^ | 0.115 | 1.72 (1.01, 2.93) ^e^ | 0.046* |
| UGI | 28.6 | 0.0 | 72.7 | n/a | n/a | 2.44 (0.87, 6.84) ^e^ | 0.091 |
| Missing | 0 | 3 | 6 |  |  |  |  |
| CRC/UGI | 0/0 | 3/0 | 4/2 |  |  |  |  |
| PG SGA score, mean |  |  |  |  |  |  |  |
| All participants | 6.4 | 4.2 | 6.1 | -1.7 (-2.2, -1.1) | <0.001* | 1.8 (0.6, 2.9) | 0.003* |
| CRC | 5.1 | 4.2 | 5.4 | -1.3 (-1.9, -0.8) | <0.001* | 1.3 (0.2, 2.4) | 0.024* |
| UGI | 6.1 | 4.6 | 9.9 | -3.6 (-5.9, -1.3) | 0.003* | 3.7 (-0.6, 8.1) | 0.094 |
| Missing | 0 | 3 | 6 |  |  |  |  |
| CRC/UGI | 0/0 | 3/0 | 4/2 |  |  |  |  |
| **Patient reported outcomes** |  |  |  |  |  |  |  |
| Missing | 2 | 5 | 5 |  |  |  |  |
| CRC/UGI | 1/1 | 4/1 | 4/1 |  |  |  |  |
| Psychological- HADS |  |  |  |  |  |  |  |
| Anxiety, mean |  |  |  |  |  |  |  |
| All participants | 5.8 | 4.7 | 4.5 | -1.1 (-1.8, -0.3) | 0.004* | -1.3 (-2.2, -0.4) | 0.004* |
| CRC | 6.2 | 5.0 | 4.5 | -1.3 (-2.1, -0.5) | <0.001* | -1.6 (-2.6, -0.6) | <0.001* |
| UGI | 4.0 | 3.5 | 4.3 | -0.1 (-1.8, 1.7) | 0.941 | 0.2 (-2.2, 2.5) | 0.893 |
| Depression, mean |  |  |  |  |  |  |  |
| All participants | 4.1 | 3.5 | 4.4 | -0.5 (-1.2, 0.2) | 0.174 | 0.4 (-0.8, 1.5) | 0.528 |
| CRC | 3.9 | 3.3 | 4.2 | -0.6 (-1.3, 0.1) | 0.085 | 0.4 (-0.8, 1.6) | 0.491 |
| UGI | 5.1 | 4.7 | 5.2 | -0.1 (-2.8, 2.5) | 0.920 | 0.1 (-3.3, 3.4) | 0.967 |
| Quality of life- EORTC QLQ-C30 |  |  |  |  |  |  |  |
| Global health status, mean ^b^ |  |  |  |  |  |  |  |
| All participants | 65.2 | 71.8 | 64.2 | 6.2 (1.8, 10.7) | 0.006* | -1.7 (-8.0, 4.5) | 0.583 |
| CRC | 63.4 | 72.3 | 64.1 | 8.4 (3.7, 13.2) | <0.001* | 0.0 (-6.6, 6.6) | 0.997 |
| UGI | 73.7 | 68.9 | 64.4 | -5.0 (-15.9, 6.0) | 0.375 | -10.3 (-27.7, 7.2) | 0.249 |
| Physical functioning, mean ^b^ |  |  |  |  |  |  |  |
| All participants | 85.2 | 88.4 | 78.3 | 2.9 (-0.1, 5.8) | 0.055 | -7.1 (-12.1, -2.1) | 0.005* |
| CRC | 86.6 | 89.5 | 78.6 | 2.9 (-0.5, 6.4) | 0.099 | -8.2 (-13.7, -2.7) | 0.004* |
| UGI | 79.0 | 82.4 | 77.0 | 2.5 (-2.0, 7.0) | 0.271 | -1.2 (-13.0, 10.6) | 0.839 |
| Role functioning, mean ^b^ |  |  |  |  |  |  |  |
| All participants | 85.8 | 87.1 | 73.4 | 1.0 (-3.8, 5.8) | 0.680 | -12.6 (-20.3, -5.0) | 0.001* |
| CRC | 85.2 | 87.8 | 74.7 | 2.5 (-3.0, 8.0) | 0.379 | -10.9 (-19.5, -2.4) | 0.012* |
| UGI | 88.5 | 83.3 | 66.7 | -6.5 (-14.5, 1.5) | 0.113 | -20.1 (-38.1, -2.1) | 0.029* |
| Emotional functioning, mean ^b^ |  |  |  |  |  |  |  |
| All participants | 79.8 | 85.7 | 84.3 | 5.6 (1.7, 9.4) | 0.005* | 4.2 (-1.3, 9.8) | 0.132 |
| CRC | 78.4 | 84.4 | 83.9 | 6.3 (1.8, 10.8) | 0.006* | 5.3 (-0.7, 11.2) | 0.081 |
| UGI | 86.5 | 92.4 | 86.4 | 3.1 (-3.2, 9.4) | 0.342 | -0.7 (-16.0, 14.6) | 0.932 |
| Cognitive functioning, mean ^b^ |  |  |  |  |  |  |  |
| All participants | 84.7 | 91.0 | 88.6 | 6.0 (2.1, 10.0) | 0.003* | 3.0 (-2.3, 8.3) | 0.267 |
| CRC | 86.0 | 92.6 | 89.9 | 6.5 (2.4, 10.5) | 0.002* | 3.3 (-2.6, 9.1) | 0.277 |
| UGI | 78.2 | 83.3 | 81.8 | 3.8 (-9.2, 16.7) | 0.568 | 1.7 (-10.9, 14.2) | 0.796 |
| Social functioning, mean ^b^ |  |  |  |  |  |  |  |
| All participants | 81.6 | 86.3 | 77.6 | 4.6 (-0.2, 9.4) | 0.059 | -4.1 (-11.0, 2.7) | 0.236 |
| CRC | 81.5 | 87.8 | 79.2 | 6.4 (1.5, 11.2) | 0.010* | -2.9 (-9.8, 4.1) | 0.418 |
| UGI | 82.1 | 78.8 | 69.7 | -4.1 (-19.4, 11.2) | 0.600 | -10.3 (-33.1, 12.5) | 0.377 |
| Fatigue, mean ^c^ |  |  |  |  |  |  |  |
| All participants | 23.9 | 19.9 | 30.2 | -3.8 (-7.5, -0.2) | 0.039* | 6.4 (0.7, 12.2) | 0.029* |
| CRC | 23.1 | 18.8 | 28.0 | -4.2 (-8.2, -0.2) | 0.040* | 5.4 (-0.5, 11.3) | 0.072 |
| UGI | 27.4 | 25.3 | 41.4 | -2.1 (-11.1, 6.8) | 0.639 | 11.8 (-7.1, 30.8) | 0.221 |
| Nausea/vomiting, mean ^c^ |  |  |  |  |  |  |  |
| All participants | 2.4 | 3.2 | 3.2 | 0.7 (-1.5, 2.9) | 0.540 | 0.7 (-2.1, 3.5) | 0.624 |
| CRC | 2.7 | 3.6 | 2.4 | 0.8 (-1.8, 3.4) | 0.546 | -0.4 (-2.7, 1.8) | 0.723 |
| UGI | 1.3 | 1.5 | 7.6 | 0.1 (-3.9, 4.1) | 0.969 | 6.2 (-6.2, 18.6) | 0.325 |
| Pain, mean ^c^ |  |  |  |  |  |  |  |
| All participants | 14.4 | 12.2 | 23.9 | -1.5 (-5.5, 2.6) | 0.481 | 9.7 (3.7, 15.7) | 0.001* |
| CRC | 12.9 | 11.3 | 21.4 | -1.0 (-5.5, 3.5) | 0.652 | 8.9 (2.3, 15.6) | 0.009* |
| UGI | 21.8 | 16.7 | 36.4 | -4.2 (-14.9, 6.4) | 0.435 | 12.3 (-2.2, 26.8) | 0.096 |
| Dyspnoea, mean ^c^ |  |  |  |  |  |  |  |
| All participants | 13.8 | 10.0 | 11.4 | -4.5 (-8.8, -0.2) | 0.038* | -3.0 (-7.9, 1.9) | 0.227 |
| CRC | 12.9 | 8.3 | 11.9 | -5.1 (-9.8, -0.3) | 0.036* | -1.5 (-6.7, 3.7) | 0.583 |
| UGI | 17.9 | 18.2 | 9.1 | -1.6 (-11.9, 8.7) | 0.766 | -11.0 (-23.7, 1.8) | 0.092 |
| Insomnia, mean ^c^ |  |  |  |  |  |  |  |
| All participants | 23.6 | 21.9 | 22.4 | -1.7 (-8.5, 5.1) | 0.625 | -0.9 (-9.2, 7.5) | 0.836 |
| CRC | 23.7 | 22.0 | 22.6 | -1.7 (-8.0, 4.7) | 0.605 | -0.7 (-10.0, 8.6) | 0.883 |
| UGI | 23.1 | 21.2 | 21.2 | -1.8 (-28.7, 25.0) | 0.894 | -1.8 (-21.4, 17.7) | 0.855 |
| Appetite loss, mean ^c^ |  |  |  |  |  |  |  |
| All participants | 13.3 | 9.5 | 18.9 | -3.9 (-8.5, 0.7) | 0.101 | 5.5 (-2.0, 12.9) | 0.151 |
| CRC | 12.9 | 8.9 | 16.1 | -4.3 (-8.8, 0.3) | 0.065 | 3.2 (-4.8, 11.1) | 0.434 |
| UGI | 15.4 | 12.1 | 33.3 | -1.7 (-17.8, 14.4) | 0.839 | 16.7 (-3.4, 36.8) | 0.103 |
| Constipation, mean ^c^ |  |  |  |  |  |  |  |
| All participants | 17.3 | 10.9 | 8.5 | -6.5 (-12.8, -0.2) | 0.042* | -9.0 (-16.9, -1.2) | 0.023* |
| CRC | 18.8 | 11.3 | 7.7 | -7.5 (-15.0, 0.0) | 0.049* | -11.3 (-20.4, -2.3) | 0.014* |
| UGI | 10.3 | 9.1 | 12.1 | -2.9 (-8.4, 2.7) | 0.308 | 3.1 (-7.2, 13.4) | 0.555 |
| Diarrhoea, mean ^c^ |  |  |  |  |  |  |  |
| All participants | 20.4 | 15.4 | 13.9 | -5.6 (-11.5, 0.4) | 0.068 | -7.4 (-14.8, 0.0) | 0.050* |
| CRC | 20.4 | 17.3 | 12.5 | -3.4 (-9.4, 2.6) | 0.263 | -8.6 (-16.7, -0.5) | 0.038* |
| UGI | 20.5 | 6.1 | 21.2 | -16.8 (-35.6, 1.9) | 0.078* | -1.2 (-18.7, 16.4) | 0.896 |
| Financial difficulties, mean ^c^ |  |  |  |  |  |  |  |
| All participants | 12.9 | 9.5 | 11.4 | -2.6 (-6.7, 1.5) | 0.215 | -0.9 (-6.4, 4.6) | 0.754 |
| CRC | 11.8 | 8.9 | 10.7 | -2.8 (-7.3, 1.8) | 0.235 | -0.2 (-6.7, 6.2) | 0.943 |
| UGI | 17.9 | 12.1 | 15.2 | -0.9 (-9.9, 8.1) | 0.843 | -5.1 (-12.8, 2.7) | 0.203 |
| Distress |  |  |  |  |  |  |  |
| Distress thermometer, mean |  |  |  |  |  |  |  |
| All participants | 7.3 | 8.1 | 8.4 | 0.7 (0.1, 1.2) | 0.016* | 1.1 (0.3, 1.8) | 0.006* |
| CRC | 7.2 | 8.1 | 8.5 | 0.9 (0.3, 1.4) | 0.002* | 1.2 (0.4, 2.0) | 0.003* |
| UGI | 7.9 | 8.1 | 8.2 | -0.2 (-2.0, 1.7) | 0.872 | 0.2 (-1.8, 2.2) | 0.832 |
| FACIT F (fatigue) |  |  |  |  |  |  |  |
| Fatigue, mean |  |  |  |  |  |  |  |
| All participants | 43.1 | 44.1 | 40.7 | 1.0 (-0.5, 2.5) | 0.207 | -2.5 (-5.0, 0.0) | 0.048* |
| CRC | 43.3 | 44.8 | 41.6 | 1.6 (-0.1, 3.2) | 0.061* | -1.9 (-4.2, 0.4) | 0.097 |
| UGI | 42.0 | 40.5 | 35.9 | -1.8 (-5.6, 2.0) | 0.358 | -5.8 (-15.6, 4.1) | 0.252 |
| ADL |  |  |  |  |  |  |  |
| ADL, mean |  |  |  |  |  |  |  |
| All participants | 6.0 | 6.0 | 5.8 | 0.0 (-0.1, 0.0) | 0.191 | -0.1 (-0.3, 0.0) | 0.069 |
| CRC | 6.0 | 5.9 | 5.9 | 0.0 (-0.1, 0.0) | 0.257 | -0.1 (-0.2, 0.1) | 0.374 |
| UGI | 6.0 | 6.0 | 5.5 | 0.0 (-0.4, 0.4) | 1.000 | -0.5 (-1.0, -0.1) | 0.016* |
| Sarcopenia screen |  |  |  |  |  |  |  |
| SARC F Score, mean |  |  |  |  |  |  |  |
| All participants | 0.6 | 0.5 | 0.9 | -0.1 (-0.2, 0.1) | 0.485 | 0.3 (-0.1, 0.6) | 0.109 |
| CRC | 0.5 | 0.5 | 0.8 | -0.1 (-0.2, 0.1) | 0.408 | 0.2 (0.0, 0.5) | 0.102 |
| UGI | 0.9 | 0.9 | 1.4 | 0.0 (-0.3, 0.3) | 0.867 | 0.4 (-0.9, 1.6) | 0.573 |
| Missing | 2 | 4 | 5 |  |  |  |  |
| CRC/UGI | 1/1 | 3/1 | 4/1 |  |  |  |  |
| Self-reported exercise modified Godin Shephard Leisure Time Physical Activity Questionnaire | |  |  |  |  |  |  |
| Light intensity (mins/week), mean |  |  |  |  |  |  |  |
| All participants | 119.8 | 85.9 | 95.5 | -33.5 (-82.6, 15.6) | 0.181 | -24.4 (-72.5, 23.8) | 0.321 |
| CRC | 107.7 | 85.3 | 97.2 | -22.4 (-75.7, 30.9) | 0.410 | -10.5 (-59.7, 38.8) | 0.677 |
| UGI | 177.7 | 89.1 | 87.3 | -95.3 (-222.9, 32.4) | 0.144 | -99.4 (-249.7, 51.0) | 0.195 |
| Moderate intensity (mins/week), mean |  |  |  |  |  |  |  |
| All participants | 53.3 | 217.0 | 75.4 | 163.2 (116.0, 210.3) | <0.001* | 22.2 (-9.4, 53.7) | 0.169 |
| CRC | 53.7 | 207.4 | 68.3 | 153.3 (99.5, 207.1) | <0.001* | 15.1 (-18.7, 48.9) | 0.380 |
| UGI | 51.5 | 267.0 | 111.4 | 210.6 (125.3, 295.8) | <0.001* | 51.5 (-34.7, 137.6) | 0.242 |
| Vigorous intensity (mins/week), mean |  |  |  |  |  |  |  |
| All participants | 19.3 | 50.8 | 3.6 | 30.5 (12.2, 48.8) | 0.001* | -17.1 (-31.7, -2.5) | 0.021* |
| CRC | 22.9 | 60.9 | 4.3 | 37.1 (15.5, 58.7) | <0.001* | -19.9 (-37.2, -2.6) | 0.025* |
| UGI | 2.3 | 0.0 | 0.0 | -2.3 (-6.8, 2.2) | 0.317 | -2.3 (-6.8, 2.2) | 0.317 |
| Resistance (mins/week), mean |  |  |  |  |  |  |  |
| All participants | 17.3 | 61.6 | 22.5 | 43.8 (32.1, 55.4) | <0.001* | 4.4 (-17.4, 26.2) | 0.691 |
| CRC | 21.0 | 62.6 | 23.0 | 41.3 (28.1, 54.4) | <0.001* | 1.4 (-24.3, 27.2) | 0.913 |
| UGI | 0.0 | 56.4 | 19.5 | 56.2 (31.7, 80.7) | <0.001* | 19.3 (-2.2, 40.7) | 0.078 |
| Missing | 2 | 6 | 5 |  |  |  |  |
| (CRC/UGI) | 1/1 | 5/1 | 4/1 |  |  |  |  |

Abbreviations:

CRC, colorectal cancer; UGI, upper gastrointestinal; kg, kilogram, BMI, body mass index, PG-SGA, Patient-Generated Subjective Global Assessment; HADS, Hospital Anxiety and Depression Scale; EORTC QLQ-C30, European Organization for the Research and Treatment of Cancer Quality of Life Questionnaire; FACIT*-*F, Functional Assessment of Chronic Illness Therapy—Fatigue; ADL, activities daily living; SARC-F, Strength, assistance with walking, rising from a chair, climbing stairs, and falls.

^a^ Derived with imputation of missing data and adjusted for confounders, including age, gender and surgeon. A Gaussian distribution estimated mean differences for continuous outcomes, and a Poisson distribution estimated relative risks for dichotomous outcomes.

^b^ Higher scores indicate a better quality of life and a higher level of functioning.

^c^ Higher scores indicate a worse quality of life and a higher level of symptoms.

^d^ Indicates >30% of participants missing.

^e^ Relative risks (RR).

*Two-sided, significant at *p* < 0.05

**Supplementary Table 4:** Surgical characteristics and outcomes.

| **Operative outcomes** | **Prehab-GI N=72** | **Historical control group (2019) N= 123** | **Adjusted mean difference ^a^ (95% CI)** | **p-value** | |
| --- | --- | --- | --- | --- | --- |
| Cancer type, n (%) |  |  |  | |  |
| Colorectal | 60 (83.3) | 111 (90.2) |  | | 0.249 |
| UGI | 12 (16.7) | 12 (9.8) |  | |  |
| **Intraoperative characteristics** |  |  |  | |  |
| Laparoscopic approach, n (%) | 56 (77.8) | 100 (81.3) |  | | 0.553 |
| Type of surgery |  |  |  | |  |
| Colon | 42 (58.3) | 79 (64.2) |  | | 0.071 |
| Rectal | 18 (25.0) | 32 (26.0) |  | |  |
| Gastric | 6 (8.3) | 1 (0.8) |  | |  |
| Oesophageal | 1 (1.4) | 5 (4.1) |  | |  |
| Pancreas | 1 (1.4) | 2 (1.6) |  | |  |
| Liver | 4 (5.6) | 4 (3.3) |  | |  |
| Stoma created, n (%) | 12 (16.7) | 28 (22.8) |  | | 0.309 |
| **Postoperative outcomes ^b^** |  |  |  | |  |
| Complications |  |  |  | |  |
| Total, n (%) |  |  |  | |  |
| All participants | 30 (41.7) | 52 (42.3) | RR, 1.0 (0.7, 1.4) | | 0.933 |
| CRC | 21 (35.0) | 45 (40.5) | RR, 0.8 (0.6, 1.3) | | 0.395 |
| UGI | 9 (75.0) | 7 (58.3) | RR, 1.5 (0.9, 2.6) | | 0.103 |
| Severe, Clavien Dindo grade >3, n (%) |  |  |  | |  |
| All participants | 6 (8.3) | 14 (11.4) | RR, 0.6 (0.2, 1.4) | | 0.226 |
| CRC | 3 (5.0) | 11 (8.9) | RR, 0.5 (0.2, 1.8) | | 0.310 |
| UGI | 3 (25.0) | 3 (25.0) | RR, 0.5 (0.1, 2.1) | | 0.339 |
| Length of hospital stay (days), mean |  |  |  | |  |
| All participants | 6.9 | 8.8 | MD, -2.1 (-3.6, -0.5) | | 0.010* |
| CRC | 5.8 | 8.6 | MD, -2.3 (-3.9, -0.8) | | 0.004* |
| UGI | 11.9 | 10.9 | MD, 2.0 (-6.3, 10.4) | | 0.639 |
| Unplanned ICU admissions, n (%) |  |  |  | |  |
| All participants | 4 (5.6) | 8 (6.5) | RR, 0.8 (0.7, 2.7) | | 0.791 |
| CRC | 2 (3.3) | 8 (7.2) | RR, 0.5 (0.1, 2.2) | | 0.343 |
| UGI | 2 (16.7) | 0 (0) | RR, 4.3 (0.2, 82.3) | | 0.329 |
| Readmissions in 30-days, n (%) |  |  |  | |  |
| All participants | 6 (8.3) | 9 (7.3) | RR, 1.1 (0.4, 3.1) | | 0.797 |
| CRC | 5 (8.3) | 7 (6.3) | RR, 1.3 (0.4, 3.9) | | 0.641 |
| UGI | 1 (8.3) | 2 (16.7) | RR, 0.5 (0.1, 4.7) | | 0.546 |

**Abbreviations:**
CRC, colorectal cancer; UGI, upper gastrointestinal; ICU, intensive care unit; MD, mean difference; RR, relative risk.
^a^ Adjustment for confounders, including age, gender and surgeon. continuous outcomes were assessed using linear regression (MD) and dichotomous variables using logistic regression (RR or χ^2^)

^b^ Measured at 30-days.

* Two-sided, significant at *p* < 0.05

**References**

1. Charlson M, Szatrowski TP, Peterson J, Gold J. Validation of a combined comorbidity index. Journal of clinical epidemiology. 1994;47(11):1245-51.

2. Aaronson NK, Ahmedzai S, Bergman B, Bullinger M, Cull A, Duez NJ, et al. The European Organization for Research and Treatment of Cancer QLQ-C30: a quality-of-life instrument for use in international clinical trials in oncology. J Natl Cancer Inst. 1993;85(5):365-76.

3. Jones CJ, Rikli RE, Beam WC. A 30-s Chair-Stand Test as a Measure of Lower Body Strength in Community-Residing Older Adults. Research Quarterly for Exercise and Sport. 1999;70(2):113-9.

4. Bohannon RW. Reference values for the timed up and go test: a descriptive meta-analysis. J Geriatr Phys Ther. 2006;29(2):64-8.

5. Schmidt K, Vogt L, Thiel C, Jäger E, Banzer W. Validity of the six-minute walk test in cancer patients. Int J Sports Med. 2013;34(7):631-6.

6. Katz S, Downs TD, Cash HR, Grotz RC. Progress in development of the index of ADL. Gerontologist. 1970;10(1):20-30.

7. Ottery FD. Patient-Generated Subjective Global Assessment. In: McCallum PD, Polisena CG, eds. The Clinical Guide to Oncology Nutrition. . Chicago: The American Dietetic Association,. 2000:11–23. .

8. Godin G. The Godin-Shephard Leisure Time Physical Activity Questionnaire. . Health Fit J Can. 2011;4:18-22.

9. Cella D. The Functional Assessment of Cancer Therapy-Anemia (FACT-An) Scale: a new tool for the assessment of outcomes in cancer anemia and fatigue. Semin Hematol. 1997;34(3 Suppl 2):13-9.

10. Zigmond AS, Snaith RP. The hospital anxiety and depression scale. Acta psychiatrica Scandinavica. 1983;67(6):361-70.

11. Malmstrom M, Ivarsson B, Johansson J, Klefsgard R. Long-term experiences after oesophagectomy/gastrectomy for cancer--a focus group study. Int J Nurs Stud. 2013;50(1):44-52.

12. Panel PDPG. NCCN practice guidelines for the management of psychosocial distress. National Comprehensive Cancer Network. Oncology (Williston Park). 1999;13(5A):113-47.

13. Dindo D, Demartines N, Clavien PA. Classification of surgical complications: a new proposal with evaluation in a cohort of 6336 patients and results of a survey. Ann Surg. 2004;240(2):205-13.
